# Integrated analysis of single-cell and bulk RNA sequencing data reveals a myeloid cell-related regulon predicting neoadjuvant immunotherapy response across cancers

**DOI:** 10.1186/s12967-024-05123-9

**Published:** 2024-05-21

**Authors:** Hong Liu, Xiaoxian Sima, Bijing Xiao, Haimiti Gulizeba, Shen Zhao, Ting Zhou, Yan Huang

**Affiliations:** Department of Medical Oncology, State Key Laboratory of Oncology in South China, Collaborative Innovation Center for Cancer Medicine, Sun Yat-sen University Cancer Center, 651 Dongfeng Road East, Guangzhou, 510060 P. R. China

**Keywords:** Single cell, Immunotherapy, Transcription factor regulons, Myeloid cells

## Abstract

**Background:**

Immunotherapy has brought about a paradigm shift in the treatment of cancer. However, the majority of patients exhibit resistance or become refractory to immunotherapy, and the underlying mechanisms remain to be explored.

**Methods:**

Sing-cell RNA sequencing (scRNA‑seq) datasets derived from 1 pretreatment and 1 posttreatment achieving pathological complete response (pCR) patient with lung adenocarcinoma (LUAD) who received neoadjuvant immunotherapy were collected, and pySCENIC was used to find the gene regulatory network (GRN) between cell types and immune checkpoint inhibitor (ICI) response. A regulon predicting ICI response was identified and validated using large‑scale pan-cancer data, including a colorectal cancer scRNA‑seq dataset, a breast cancer scRNA‑seq dataset, The Cancer Genome Atlas (TCGA) pan-cancer cohort, and 5 ICI transcriptomic cohorts. Symphony reference mapping was performed to construct the myeloid cell map.

**Results:**

Thirteen major cluster cell types were identified by comparing pretreatment and posttreatment patients, and the fraction of myeloid cells was higher in the posttreatment group (19.0% vs. 11.8%). A PPARG regulon (containing 23 target genes) was associated with ICI response, and its function was validated by a colorectal cancer scRNA‑seq dataset, a breast cancer scRNA‑seq dataset, TCGA pan-cancer cohort, and 5 ICI transcriptomic cohorts. Additionally, a myeloid cell map was developed, and cluster I, II, and III myeloid cells with high expression of PPARG were identified. Moreover, we constructed a website called PPARG (https://pparg.online/PPARG/ or http://43.134.20.130:3838/PPARG/), which provides a powerful discovery tool and resource value for researchers.

**Conclusions:**

The PPARG regulon is a predictor of ICI response. The myeloid cell map enables the identification of PPARG subclusters in public scRNA-seq datasets and provides a powerful discovery tool and resource value.

**Supplementary Information:**

The online version contains supplementary material available at 10.1186/s12967-024-05123-9.

## Background

Immune checkpoint inhibitors (ICIs) are capable of disrupting immune surveillance subversion by cancer cells and have dramatically altered cancer treatment paradigms [1]. However, as ICIs have shown activity in only a minority of patients with specific cancers [1, 2], additional therapeutic strategies to potentiate antitumour immunity have been proposed.

The advent of ICIs has introduced a transformative era in cancer treatment, delivering unprecedented clinical benefits for patients [3–5]. However, only subsets of patients with specific tumour types respond to ICIs [6, 7], and their efficacy by existing measures is limited. It is necessary to perform extensive biomarker research to optimize patient selection and develop combination strategies to overcome immune resistance. Traditional biomarker studies primarily focused on analysing RNA sequencing (RNA-seq) data [8, 9] on the assumption that cells from a given tissue were homogeneous and thus missing important cell-to-cell variability. Single-cell RNA sequencing (scRNA-seq) is a powerful technique for dissecting the complexity of solid tumours, enabling the characterization of cell diversity and heterogeneous phenotypic states in unprecedented detail [10]. The advent of scRNA-seq has enabled us to analyse gene expression at the level of individual cells, leading to the discovery of new biomarkers that demonstrate superior performance [11]. The application of scRNA-seq has revolutionized our understanding of cell states and heterogeneity within the tumour microenvironment (TME).

The TME plays an important role in tumorigenesis and drug resistance [12, 13]. Immunotherapy could alter the TME, and conversely, the TME could influence the efficacy of immunotherapy [14]. Very few studies have explored the transcriptomic characteristics of different cell types in the TME based on scRNA-seq data and their correlation with the efficacy of pan-cancer neoadjuvant immunotherapy. A single-cell transcriptome atlas of the TME could improve the understanding of cell types and ICI responses.

Myeloid cells encompass a diverse population of innate immune cells, which can be broadly classified into subgroups including monocytes, macrophages, granulocytes (comprising neutrophils, basophils, eosinophils, and mast cells), and dendritic cells. For several decades, extensive research has focused on utilizing targeted strategies against specific subsets of myeloid cells as a potential therapeutic approach in cancers [15, 16]. Nevertheless, the presence of developmental and functional heterogeneity within myeloid cell subsets has posed significant challenges to the implementation of this approach. Furthermore, the absence of consistent markers across mouse and human myeloid cell subsets adds further complexity to identifying clinically significant targets and translating preclinical discoveries into a clinical context.

Gene regulatory networks (GRNs) [17] are intricate regulatory circuits formed through the dynamic interactions of chromatin, transcription factors, and genes, including the interplay between transcription factors (TFs) and target genes. With the emergence of single-cell technologies, the application of GRNs across different cell types, differentiation trajectories and conditions is possible.

Thus, in this study, we revealed and verified the GRNs between TFs and target genes and their association with ICI outcomes using 3 scRNA-seq datasets, pan-cancer transcriptomic data, and 5 independent ICI cohorts. Our findings uncovered the potential of the peroxisome proliferator-activated receptor-γ (PPARG) regulon for predicting ICI outcomes across multiple cancer types. With the popularity of single-cell sequencing, predicting therapeutic efficacy has become feasible. Therefore, we constructed a predictive map based on the PPARG regulon and developed corresponding scripts.

## Methods

### Single-cell data collection

Lung cancer scRNA-seq data (GSE207422) [18] containing paired bulk RNA-seq data and colorectal cancer (CRC) scRNA-seq data GSE205506 [19] were downloaded from the Gene Expression Omnibus (GEO) (https://www.ncbi.nlm.nih.gov/geo/) database for analysis. Breast cancer (BRCA) scRNA-seq data [20] were downloaded from lambrechtslab data access (vib.be) for external validation. Specifically, we extracted pre- and posttreatment data from lung adenocarcinoma (LUAD) patients and CRC patients and nonresponse and response data after anti-programmed cell death protein 1 (PD-1) treatment from early-diagnosed BRCA patients who received neoadjuvant immunotherapy.

### Pan-cancer bulk RNA‑seq datasets

The bulk RNA-seq datasets GSE207422 [18], GSE126044 [21], GSE135222 [22, 23], Orient-11 [24], and PRJEB23709 [25] and The Cancer Genome Atlas (TCGA) pan-cancer bulk RNA-seq data [7] were downloaded for validation.

GSE207422, GSE126044 and GSE135222 were downloaded from GEO (https://www.ncbi.nlm.nih.gov/geo/). The TCGA pan-cancer cohort was downloaded from the UCSC Xena data portal (https://xenabrowser.net). PRJEB23709 was downloaded from The European Nucleotide Archive (ENA) (ENA Browser (ebi.ac.uk)).

### Raw data processing and quality control

Unique molecular identifier (UMI) matrices for each sample were obtained. Normalization was performed using the Seurat package (version 3.2.2) [26] in R software (version 4.3.1), dividing the UMI counts of each gene by the total UMI count of each cell and scaling by 1 × 10^4^ for computational efficiency. All normalized data were log2-transformed. Four quality control measures were used, and the exclusion criteria were as follows: (1) < 500 expressed genes, (2) > 20% UMIs of mitochondria genes, (3) > 50% UMIs of ribosome genes, and (4) housekeeping score (defined as the sum of the UMIs of three canonical housekeeping genes: ACTB, GAPDH and MALAT1) < 1. To exclude data from droplets containing more than one cell, doublet detection and removal were performed using Scrublet (version 0.2.1) [27]. The Harmony algorithm was performed to remove the batch effect [28]. An expected doublet rate parameter of 0.025 was used, and doublet score thresholds were chosen manually to divide putative singlet and neotypic doublet modes in the score distribution. Predicted doublets were then removed from gene-barcode matrices.

### Data integration, unsupervised clustering, and cell type annotation

Principal component analysis (PCA) was performed on the integration-transformed expression matrix using the RunPCA function, and the first 20 principal components (PCs) were used in the FindNeighbors function. The resolution parameters of the FindClusters function were different for different cell types, with 0.6 for all cells. Uniform manifold approximation and projection (UMAP) was performed for visualization in two dimensions using the RunUMAP function with the same PCs and other default parameters. Major cell lineages were assigned to each cluster of cells using the abundance of canonical marker genes, and marker genes for each cluster were found using the FindAllMarkers function with the parameter “min.pct = 0.25”.

### Gene set variation analysis (GSVA)

To score each sample with the gene list of the PPARG regulon, we applied GSVA using standard settings, as implemented in the GSVA R package (version 1.32.0), as described previously [29]. The gene set of PPARG regulon was identified by using pySCENIC analysis.

### Cellular fraction calculation

For each sample, we calculated the cellular fraction for each major cell lineage (T cells, B cells, myeloid cells, etc.), and for the subpopulations of major cell lineages, cellular proportions were calculated by the fractions in corresponding major immune lineages. Notably, the samples that had fewer than 10 cells in a major lineage were removed to perform downstream statistical tests. The significance of differences among response groups for the fractions was compared using a one-sided unpaired Wilcoxon rank sum test, and the P values were adjusted by the false discovery rate (FDR) method for multiple parallel tests.

### pySCENIC analysis

Single-cell regulatory network inference and clustering (SCENIC) [30] contains three main steps, including coexpression analysis, target gene motif enrichment analysis, and regulon activity evaluation. Briefly, in the first step, coexpression modules between transcription factors and candidate target genes were inferred with GENIE3. Each module consists of a transcription factor together with its predicted targets, based purely on coexpression. In the second step, each coexpressed module was analysed with RcisTarget to identify enriched motifs; only modules and targets for which the motif of the TF was enriched were retained. Each TF together with its potential direct targets is a regulon. In the third step, the activity of each regulon in each cell was evaluated using AUCell, which calculates the area under the recovery curve. The AUCell scores are used to generate the regulon activity matrix. This matrix can be binarized by setting an AUC threshold for each regulon, which will determine in which cell the regulon is “active”. The regulon activity matrix can be used to cluster the cells and thereby identify cell types and states based on the shared activity of a regulatory subnetwork. pySCENIC is a lightning-fast Python implementation of the SCENIC pipeline. As described [31], activated regulons in each cell type were analysed using pySCENIC [30] with a raw count matrix as input. The coexpression network was calculated by GRNboost, and the regulons were identified by RcisTarget. The regulon activity for each cell was scored by AUCell.

### Regulon module analysis

Regulon modules were identified based on the Connection Specificity Index (CSI) [32], which is a context-dependent measure for identifying specific associating partners. The evaluation of CSI involves two steps. First, the Pearson correlation coefficient (PCC) of activity scores was evaluated for each pair of regulons. Next, for a fixed pair of regulons, A and B, the corresponding CSI is defined as the fraction of regulons whose PCC with A and B is lower than the PCC between A and B.

Hierarchical clustering with Euclidean distance was performed based on the CSI matrix to identify different regulon modules. We also used CSI > 0.7 as a cut-off to build the regulon association network to investigate the relationships among different regulons. For each regulon module, its activity score associated with a cell type is defined as the average of the activity scores of its regulon members in all cells within this cell type. Then, the top ranked cell types are identified for each module.

### Reference mapping

Myeloid cells were purified with a set of markers (“CD14+”, “FCER1A-”) by the scGate R package (version 1.4.1) [33]. Myeloid cell reference atlas mapping was performed using the Symphony R package (version 0.1.1) [34].

### Survival analysis

The patients were divided into high and low PPARG regulon scores by the median value, and Kaplan–Meier survival curves with the cumulative number of events table and log-rank test were plotted by the survminer (version 0.4.8) and survival (version 3.5.5) R packages.

### Statistics

**Fig. 1:**
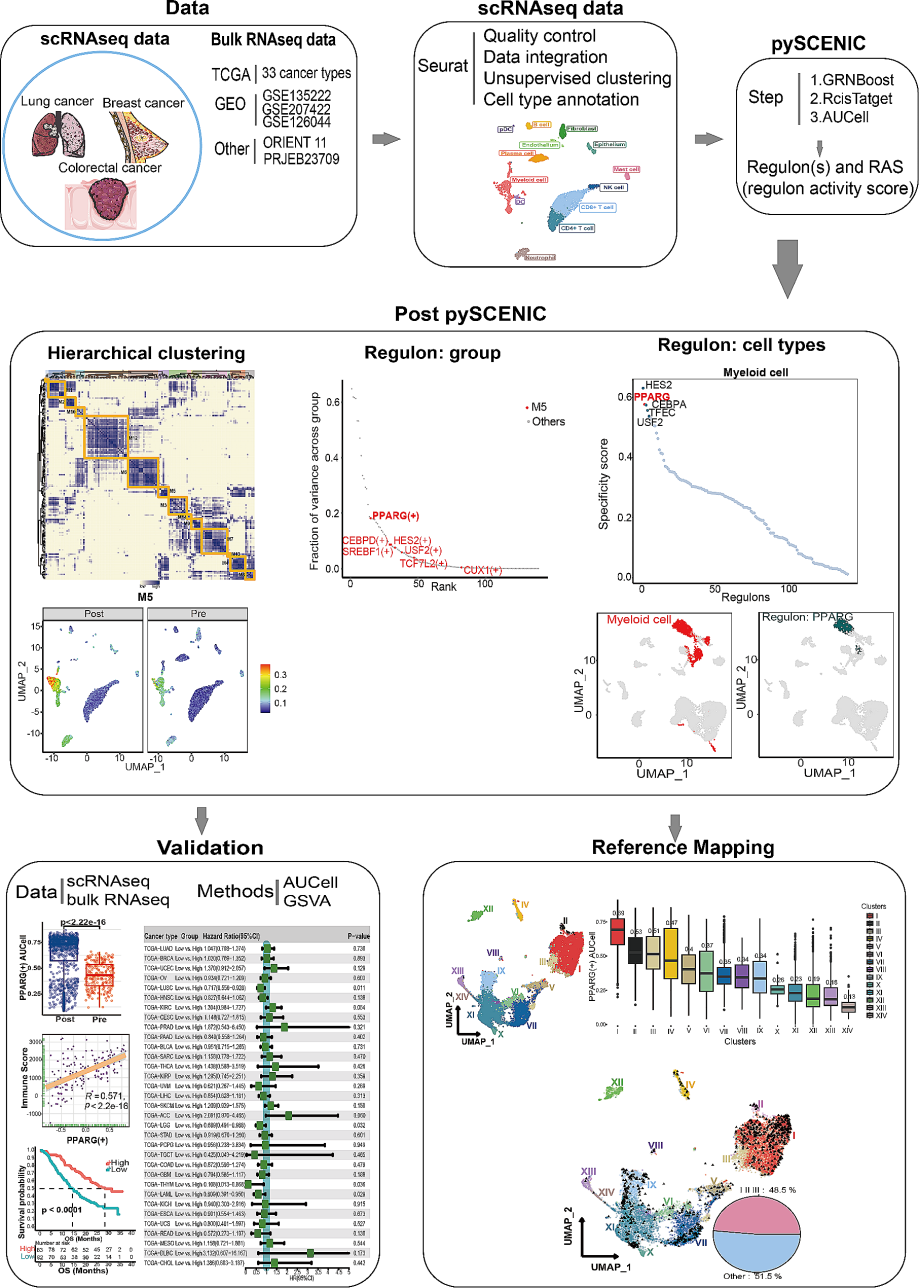
A flowchart showed the overall idea of this study

All statistical analyses and presentations were performed using R software (version 4.3.1) and Python (version 3.10.11). All statistical tests used are defined in the figure legends. Statistical significance was set at P or adjusted *P* < 0.05. A flowchart for the overall idea of this study was displayed in Fig. 1.

## Results

### Single-cell sequence analysis and cell type identification

**Fig. 2:**
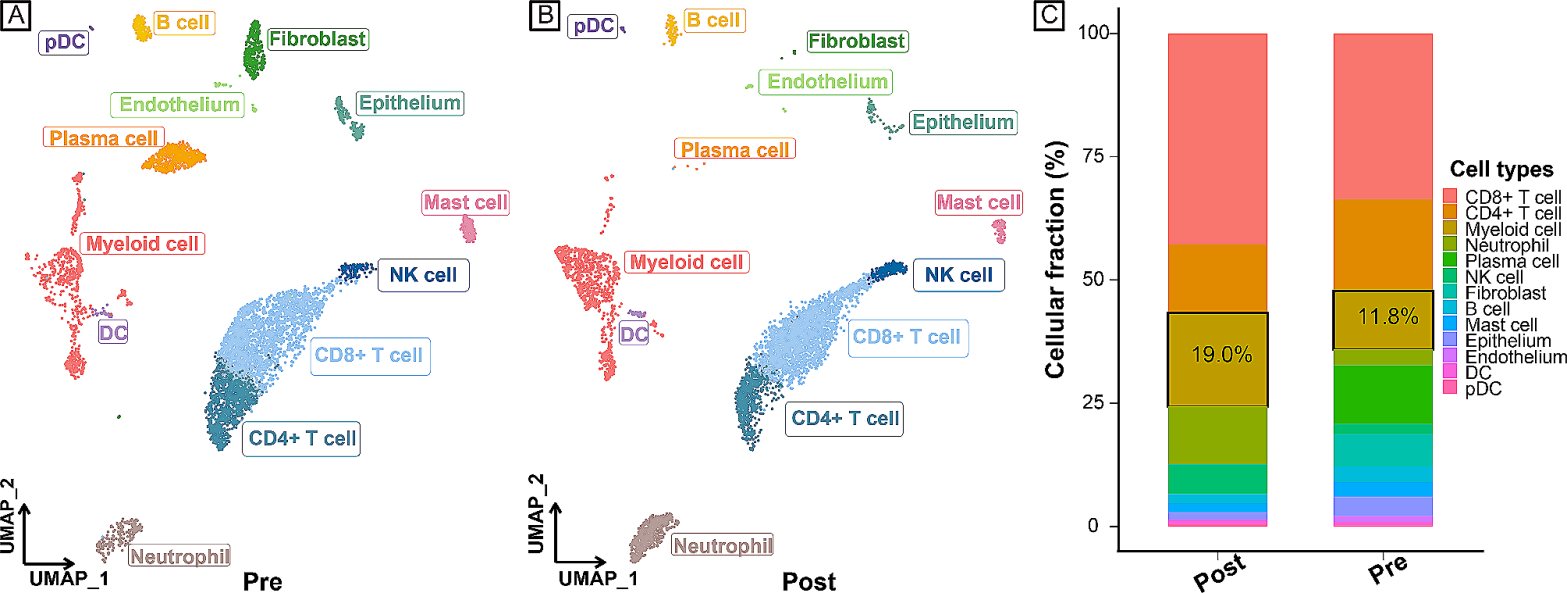
Identifying infiltrated cell types. **A**, **B** UMAP plot of single cells profiled in the present work coloured by cell type. **A** (pre), Single-cell data of patient 08 before treatment (immunotherapy plus chemotherapy); **B** (post), single-cell data of patient 06 achieving pCR after treatment (immunotherapy plus chemotherapy). **C** Relative fraction of cell types. The relative contribution of each population was weighed by the number of cells and scaled to 100%. UMAP, uniform manifold approximation and projection; pCR, pathology complete response.

After quality control and removal of the batch effect (Supplementary Fig. 1) between pretreatment (PD-1 antibody combined with chemotherapy, patient 08) and posttreatment (achieving pCR, patient 06) GSE207422 scRNA-seq data, 10,441 single cells were clustered into 13 major clusters (Fig. 2A, B). Cluster-specific genes were used to annotate cell types with classic markers.

To characterize TME remodelling in response to treatment, we calculated the fraction of different cell types in pretreatment and posttreatment patients (Fig. 2C).

### Regulons are organized into combinatorial modules

We applied SCENIC to identify key regulons. SCENIC can simultaneously reconstruct gene regulatory networks and identify cell states from scRNA-seq data. Significantly active regulons were identified.

SCENIC links cisregulatory sequence information together with RNA-seq data. SCENIC contains three main steps, including coexpression analysis, target gene motif enrichment analysis, and regulon activity evaluation. The main outcomes contain a list of regulons (each representing a TF along with a set of coexpressed and motif significantly enriched target genes) and the regulon activity scores (RAS) for each cell.

**Fig. 3:**
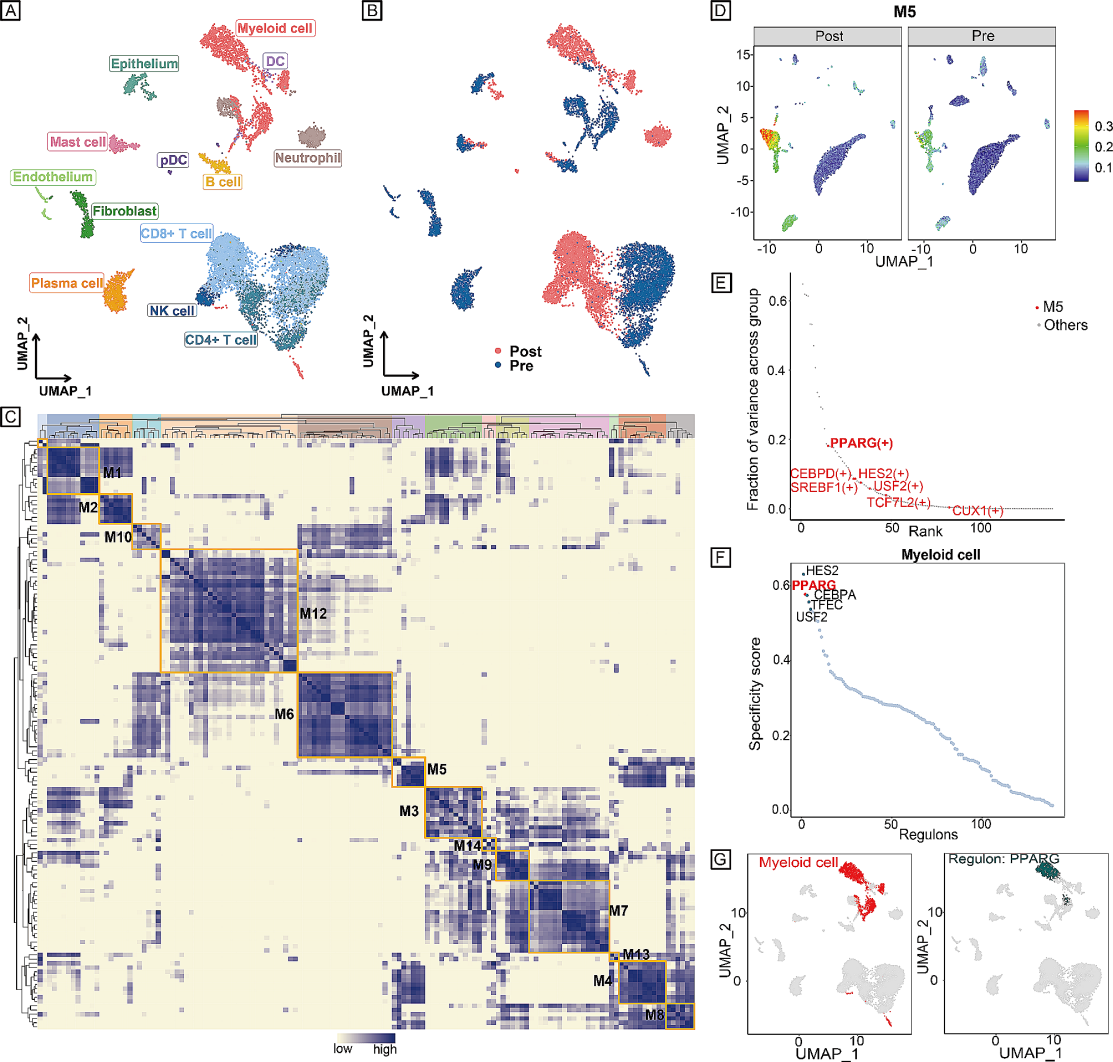
Identification of combinatorial regulon modules. **A** UMAP for all single cells based on RAS; each cell is colour-coded based on cell-type assignment. **B** UMAP for all single cells based on RAS; each cell is colour-coded based on pre/posttreatment. **C** Identified regulon modules based on the regulon CSI matrix. **D** UMAP for all single cells in module M5 based on average RAS. **E** Regulons in module M5 (red dots). **F** Rank for regulons in myeloid cells based on the RSS. **G** (left) Myeloid cells are highlighted in the UMAP (red dots); (right) Binarized RAS for the top regulon PPARG on UMAP (green dots). UMAP, uniform manifold approximation and projection; RAS, regulon activity scores; CSI, connection specificity index; RSS, regulon specificity score.

By applying the modified SCENIC approach described above, we identified 139 significant regulons containing 8839 target genes. Different cell types (Fig. 3A), as well as pre- or postneoadjuvant therapy (Fig. 3B), can be separated based on the regulon activities in the UMAP plot.

Strikingly, these 139 regulons were organized into 14 major modules (Fig. 3C). For each module, we analysed their average activity scores, and when mapping the average activity score of each module onto UMAP, we found that, comparing pre- and postneoadjuvant immunotherapy combined with chemotherapy, myeloid cells demonstrated the most significant difference in module M5 (Fig. 3D and Supplementary Fig. 2).

Next, regulons were ranked by analysing the variance components in 14 modules (Supplementary Fig. 3). In addition, by calculating the regulon specificity score (RSS), we identified cell type-specific regulons (Supplementary Fig. 4). Interestingly, the PPARG regulon (containing 23 target genes: FTL, ACP5, GRN, ASAH1, FBP1, CTSS, APOE, GLUL, SLA, TXNIP, BRI3, CD68, MSR1, VSIG4, BHLHE41, ALDH2, ALOX5, CSTB, TMBIM1, CD52, LIPA, GPNMB, and CPM) was significantly shared in module M5 and was also the myeloid cell-specific regulon (Fig. 3E–G), indicating that the PPARG regulon might influence immunotherapy.

### The PPARG regulon is a predictor of immunotherapy response

To investigate the clinical role of the PPARG regulon in the present study, we validated its function using AUCell and GSVA. According to the TCGA pan-cancer cohort, there were no significant prognostic differences between the high PPARG regulon and low PPARG regulon based on univariable Cox regression analysis (Supplementary Fig. 5), indicating that the PPARG regulon was not a purely prognostic indicator.

**Fig. 4:**
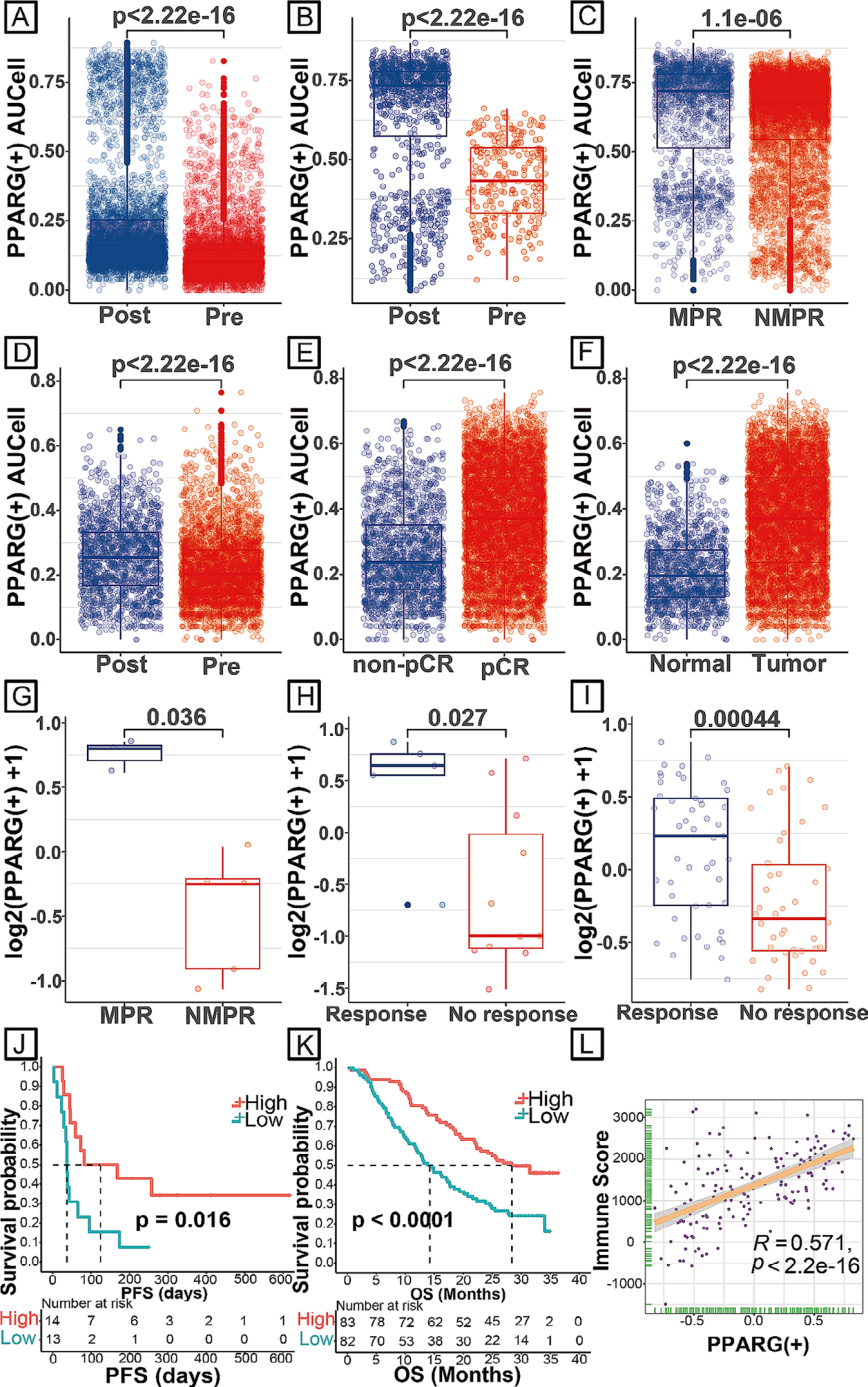
Clinical function of the PPARG regulon. **A** AUCell of the PPARG regulon between LAUD pretreatment data (GSE207422 patient 08) and posttreatment data (GSE207422 patient 06). **B** AUCell of the PPARG regulon between LAUD pretreatment data (GSE207422 patient 05) and posttreatment data (GSE207422 patient 11). **C** AUCell of the PPARG regulon between LAUD MPR and NMPR data (GSE207422). **D** AUCell of the PPARG regulon between CRC pretreatment and posttreatment data (pCR, GSE205506). **E** AUCell of the PPARG regulon between the CRC pCR and nonpCR groups (GSE205506). **F** AUCell of the PPARG regulon between CRC tumour tissues and normal tissues (GSE205506). **G** GSVA scores of the PPARG regulon between LAUD MPR and NMPR data (GSE207422). **H** GSVA scores of the PPARG regulon between NSCLC response and nonresponse data (GSE126044). **I** GSVA scores of the PPARG regulon between melanoma response and nonresponse data (PRJEB23709). **J** Kaplan–Meier survival curve of the PPARG regulon (median GSVA score) in the GSE135222 cohort. Survival curves were compared by the log-rank test. **K** Kaplan–Meier survival curve of the PPARG regulon (median GSVA score) in the Orient-11 cohort. Survival curves were compared by the log-rank test. **L** Spearman correlation between the GSVA score of the PPARG regulon and immune score in the Orient-11 cohort. LAUD, lung adenocarcinoma; MPR, major pathology response; NMPR, non-major pathology response; CRC, colorectal cancer; pCR, pathology complete response; GSVA, gene set variation analysis; NSCLC, non-small-cell lung cancer.

However, the score of the PPARG regulon was higher in the posttreatment (GSE207422 patient 06, neoadjuvant immunotherapy combined with chemotherapy) achieving pCR group than in the pretreatment group (GSE207422 patient 08, neoadjuvant immunotherapy combined with chemotherapy) (Fig. 4A and Supplementary Fig. 6A). The same results were observed in GSE207422 patient 05 (pretreatment) versus patient 11 (posttreatment achieving major pathology response [MPR]) (Fig. 4B and Supplementary Fig. 6B). Moreover, after treatment with neoadjuvant immunotherapy combined with chemotherapy, the AUCell of the PPARG regulon in the MPR group was higher than that of the non-MPR (NMPR) group based on GSE207422 data (Fig. 4C and Supplementary Fig. 6C). Another CRC scRNA-seq dataset (GSE205506) showed the same results. For patients achieving pCR, compared to that in the pretreatment data, the AUCell of the PPARG regulon was higher in the posttreatment data (Fig. 4D and Supplementary Fig. 6D). After neoadjuvant immunotherapy, a higher AUCell of the PPARG regulon was found in the pCR group and tumour tissues than in the nonpCR group and normal tissues, respectively (Fig. 4E, F and Supplementary Fig. 6E, F).

Validation was further performed by using a series of bulk RNA-seq data (GSE207422, GSE126044 and PRJEB23709; Fig. 4G–I). In another two RNA-seq datasets for advanced non-small cell lung cancer (NSCLC) patients who were treated with immunotherapy ± chemotherapy (GSE135222 and Orient-11), a high PPARG score was associated with a better prognosis (Fig. 4J–K). Additionally, the PPARG regulon was positively correlated with the immune score (Fig. 4L).

### PPARG + myeloid cell atlas

**Fig. 5:**
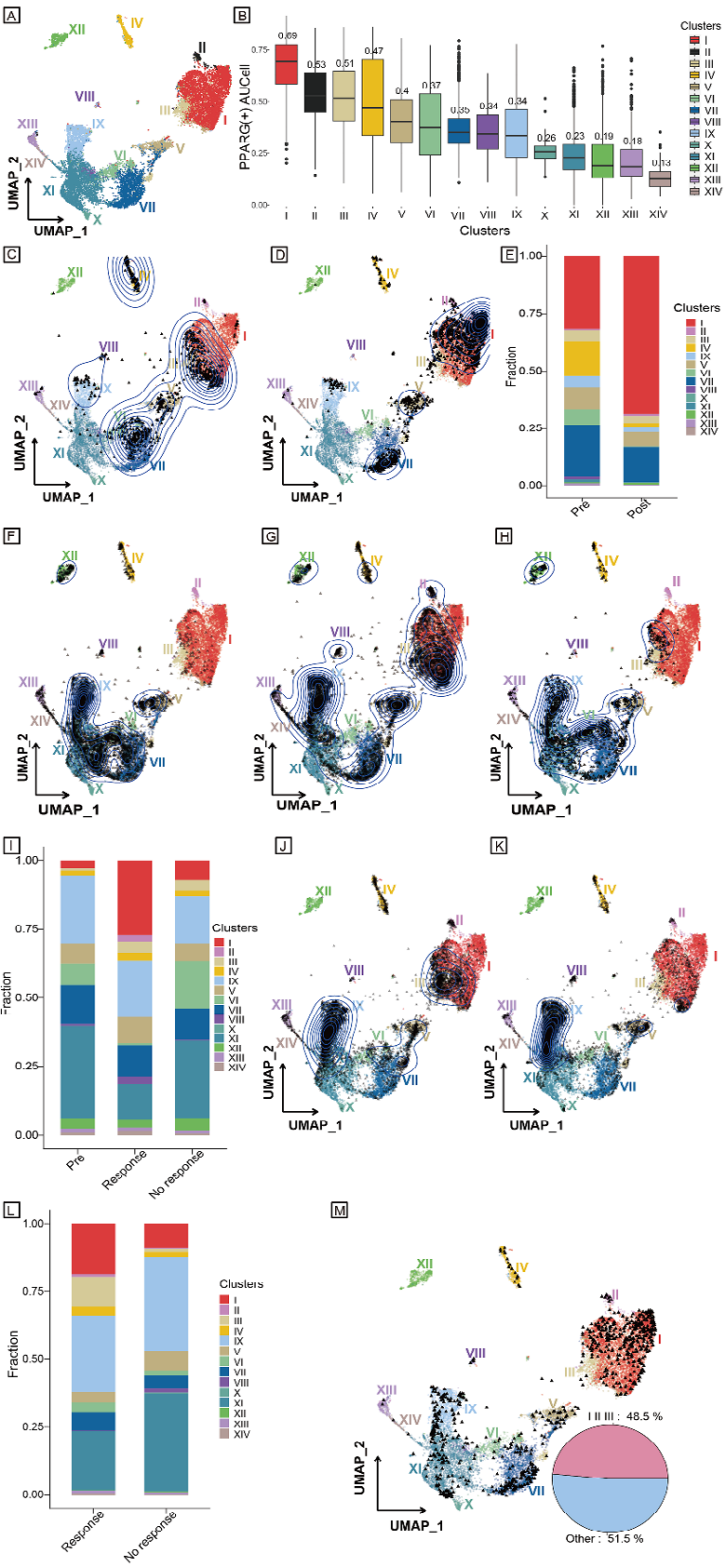
Subgroups of PPARG + myeloid cells. **A** UMAP for PPARG + myeloid cells based on AUCell from GSE207422 (LUAD samples) and GSE205506 (CRC tumour samples). **B** Box plot of 14 subgroups of PPARG + myeloid cells based on AUCell. **C** Validation UMAP for PPARG + myeloid cells mapped with pretreatment data (GSE207422 patients 05 and 08). **D** Validation UMAP for PPARG + myeloid cells mapped with posttreatment data (GSE207422 patient 06 and 11). **E** Fraction of 14 PPARG + myeloid cell subgroups between LAUD pretreatment data (GSE207422 patients 05 and 08) and posttreatment data (GSE207422 patients 06 and 11). **F** Validation UMAP for PPARG + myeloid cells mapped with CRC pretreatment data (GSE205506). **G** Validation UMAP for PPARG + myeloid cells mapped with CRC response data after treatment (GSE205506). **H** Validation UMAP for PPARG + myeloid cells mapped with CRC nonresponse data after treatment (GSE205506). **I** Fraction of 14 PPARG + myeloid cell subgroups among CRC pretreatment, response and nonresponse data after treatment (GSE205506). **J** Validation UMAP for PPARG + myeloid cells mapped with BRCA response data after treatment. **K** Validation UMAP for PPARG + myeloid cells mapped with BRCA nonresponse data after treatment. **L** Fraction of 14 PPARG + myeloid cell subgroups between BRCA response and nonresponse data after treatment. **M** Example of UMAP after symphony reference mapping and pie chart of the fractions of clusters I, II, and III. UMAP, uniform manifold approximation and projection; LUAD, lung adenocarcinoma; CRC, colorectal cancer; BRCA, breast cancer.

Next, we selected myeloid cell clusters on the basis of the expression of myeloid cell markers (“CD14+”, “FCER1A-”) by the scGate R package from GSE207422 (LUAD samples) and GSE205506 (CRC tumour samples). We obtained a total of 18,488 myeloid cells. Based on the AUCell of PPARG, we constructed a PPARG + myeloid cell atlas, and these cells were divided into 14 clusters (I-XIV) (Fig. 5A). Notably, the median AUCell of PPARG was over 0.50 in clusters I, II and III (Fig. 5B and Supplementary Table). Specifically, clusters I, II and III were dominant in the posttreatment data (GSE207422 patient 06 and patient 11) (Fig. 5C–E). Similarly, for CRC, the fraction of clusters I, II and III was larger in the treatment-response group than in the nonresponse group and pretreatment group (Fig. 5F–I).

Furthermore, external validation yielded the same results. The fraction of clusters I, II and III was higher when comparing the response group and nonresponse group of BRCA scRNA-seq data (Fig. 5J–L). Overall, clusters I, II and III were associated with immunotherapy response, and markers used to identify clusters I, II and III were provided.

In conclusion, in this study, we constructed a PPARG website (https://pparg.online/PPARG/ or http://43.134.20.130:3838/PPARG/). When a scRNA-seq data matrix is uploaded, myeloid cells are extracted. Then, AUCell scores for the PPARG regulon of each myeloid cell are calculated, and the proportions of clusters I, II and III with high expression of PPARG are identified (Fig. 5M).

## Discussion

Enhancing response rates and identifying reliable biomarkers are the major challenges for current immunotherapy. Although ICIs have been used for years, many patients are refractory to treatment.

This study involved the analysis of single-cell transcriptomes obtained from pan-cancer neoadjuvant immunotherapy data, allowing for a comprehensive assessment of the TME and an investigation into immune system and cancer responses to therapy.

We found that the fraction of myeloid cells was notably higher in the response group after neoadjuvant immunotherapy than in the pretreatment group (Fig. 1C), indicating that myeloid cells might play an important role in the immune response. Previously, single-cell analyses have also revealed the complexity of tumour-infiltrating myeloid cells in multiple different cancer types [35, 36]. However, single-cell analyses revealing treatment response-related myeloid cells across cancers are lacking. Myeloid cells are the most abundant immune components of the TME, where they have a variety of functions, ranging from immunosuppressive to immunostimulatory roles [16].

Furthermore, by using pySCENIC, the PPARG + regulon was identified in module M5 (pre vs. post, the RAS of myeloid cells was the most different), and PPARG was specifically expressed in myeloid cells (Fig. 2D–G). PPARG is a nuclear hormone receptor that regulates numerous cellular functions, including inflammation, differentiation, and tumorigenesis [37, 38]. PPARG also serves as an important regulator of anti-inflammatory activity, acting in part by antagonizing the nuclear factor-κB (NF-κB) pathway [39]. Besides, other acknowledged regulons correlated to immunotherapy response were also identified based on our methods, such as Interferon Regulatory Factor 4 (IRF4), Forkhead box protein P3 (FOXP3), and so on, indirectly reflecting the reliability of our computational method. IRF4 plays a role in regulating immune cell activation and differentiation. Manipulating IRF4 may be an important therapeutic target for reversing T cell exhaustion and TME disorders, thus promoting anti-tumor immunity [40]. FOXP3 is a key transcription factor for regulatory T cells (Tregs). Tregs are among the most abundant suppressive cells in the TME and their presence has been correlated with tumor progression, invasiveness as well as metastasis [41].

Mapping of the myeloid cell landscape together with PPARG regulon AUCell score analysis revealed that these cells could be further subcategorized into 14 clusters, in which I-III clusters had a high score over 0.5 and were indeed closely related to the efficacy of immunotherapy.

However, there were several limitations in this study. First, although we found that the PPARG regulon was a marker of immunotherapy response and that a higher PPARG AUCell score was associated with a better response, the cut-off value of the PPARG AUCell score remains to be further confirmed with large-scale scRNA-seq data. Second, some of the patients in this study received combination immunotherapy and chemotherapy. Finally, our findings await further validation by basic research and prospective study.

## Conclusions

scRNA-seq analysis combined with bulk RNA-seq analysis of pan-cancer data revealed the TME before and after neoadjuvant immunotherapy and different TME properties between good responders and poor responders. We identified that the PPARG regulon was a predictor of ICI response. Moreover, the myeloid cell atlas enabled the identification of PPARG + subclusters and provides a powerful discovery tool and resource value.

### Electronic supplementary material

Below is the link to the electronic supplementary material.

Supplementary Material 1

Supplementary Material 2

Supplementary Material 3

Supplementary Material 4

Supplementary Material 5

Supplementary Material 6

Supplementary Material 7

Supplementary Material 8

## Data Availability

The datasets analysed during the current study are available in the GEO (https://www.ncbi.nlm.nih.gov/geo/), TCGA (https://xenabrowser.net), ENA Browser (ebi.ac.uk), and lambrechtslab data access (vib.be). The data of orient-11 is available upon request from the corresponding author. The authenticity of this article has been validated by uploading the key raw data onto the Research Data Deposit (RDD) public platform (www.researchdata.org.cn).

## Abbreviations

pCR: Pathological complete response
LUAD: Lung adenocarcinoma
GRN: Gene regulatory network
ICI: Immune checkpoint inhibitor
TCGA: The Cancer Genome Atlas
RNA-seq: RNA sequencing
scRNA-seq: Single-cell RNA sequencing
TME: Tumour microenvironment
TFs: Transcription factors
PPARG: Peroxisome proliferator-activated receptor-γ
SCENIC: Single-cell regulatory network inference and clustering
PD-1: Programmed cell death protein 1
RAS: Regulon activity scores
UMAP: Uniform manifold approximation and projection
RSS: Regulon specificity score
GSVA: Gene set variation analysis
MRP: Major pathology response
NMPR: Non-MPR
CRC: Colorectal cancer
NF-κB: Nuclear factor-κB
GEO: Gene Expression Omnibus
UMI: Unique molecular identifier
PCA: Principal component analysis
PCs: Principal components
FDR: False discovery rate
CSI: Connection Specificity Index
PCC: Pearson correlation coefficient

## References

[1] Ribas A, Wolchok JD (2018). Cancer immunotherapy using checkpoint blockade. Science.

[2] Yarchoan M, Hopkins A, Jaffee EM (2017). Tumor mutational burden and response rate to PD-1 inhibition. N Engl J Med.

[3] Kruger S, Ilmer M, Kobold S, Cadilha BL, Endres S, Ormanns S, Schuebbe G, Renz BW, D’Haese JG, Schloesser H, Heinemann V, Subklewe M, Boeck S, Werner J (2019). Von Bergwelt-Baildon M. advances in cancer immunotherapy 2019 - latest trends. J Exp Clin Cancer Res CR.

[4] Mellman I, Coukos G, Dranoff G (2011). Cancer immunotherapy comes of age. Nature.

[5] Wang Y, Wang M, Wu HX, Xu RH (2021). Advancing to the era of cancer immunotherapy. Cancer Commun Lond Engl.

[6] Sharma P, Siddiqui BA, Anandhan S, Yadav SS, Subudhi SK, Gao J, Goswami S, Allison JP (2021). The Next Decade of Immune Checkpoint Therapy. Cancer Discov.

[7] Zhang Z, Wang ZX, Chen YX, Wu HX, Yin L, Zhao Q, Luo HY, Zeng ZL, Qiu MZ, Xu RH (2022). Integrated analysis of single-cell and bulk RNA sequencing data reveals a pan-cancer stemness signature predicting immunotherapy response. Genome Med.

[8] Hakimi AA, Voss MH, Kuo F, Sanchez A, Liu M, Nixon BG, Vuong L, Ostrovnaya I, Chen YB, Reuter V, Riaz N, Cheng Y, Patel P, Marker M, Reising A, Li MO, Chan TA, Motzer RJ (2019). Transcriptomic profiling of the Tumor Microenvironment reveals distinct subgroups of Clear Cell Renal Cell Cancer: data from a Randomized Phase III Trial. Cancer Discov.

[9] Ott PA, Bang YJ, Piha-Paul SA, Razak ARA, Bennouna J, Soria JC, Rugo HS, Cohen RB, O’Neil BH, Mehnert JM, Lopez J, Doi T, van Brummelen EMJ, Cristescu R, Yang P, Emancipator K, Stein K, Ayers M, Joe AK, Lunceford JK (2019). T-Cell-inflamed gene-expression Profile, programmed death Ligand 1 expression, and Tumor Mutational Burden Predict Efficacy in patients treated with Pembrolizumab Across 20 cancers: KEYNOTE-028. J Clin Oncol off J Am Soc Clin Oncol.

[10] Zhang L, Zhang Z (2019). Recharacterizing tumor-infiltrating lymphocytes by single-cell RNA sequencing. Cancer Immunol Res.

[11] Hwang B, Lee JH, Bang D (2018). Single-cell RNA sequencing technologies and bioinformatics pipelines. Exp Mol Med.

[12] Hinshaw DC, Shevde LA (2019). The Tumor Microenvironment innately modulates Cancer Progression. Cancer Res.

[13] Zhang A, Miao K, Sun H, Deng CX (2022). Tumor heterogeneity reshapes the tumor microenvironment to influence drug resistance. Int J Biol Sci.

[14] Binnewies M, Roberts EW, Kersten K, Chan V, Fearon DF, Merad M, Coussens LM, Gabrilovich DI, Ostrand-Rosenberg S, Hedrick CC, Vonderheide RH, Pittet MJ, Jain RK, Zou W, Howcroft TK, Woodhouse EC, Weinberg RA, Krummel MF (2018). Understanding the tumor immune microenvironment (TIME) for effective therapy. Nat Med.

[15] Mantovani A, Allavena P, Marchesi F, Garlanda C (2022). Macrophages as tools and targets in cancer therapy. Nat Rev Drug Discov.

[16] Goswami S, Anandhan S, Raychaudhuri D, Sharma P (2023). Myeloid cell-targeted therapies for solid tumours. Nat Rev Immunol.

[17] Badia-I-Mompel P, Wessels L, Müller-Dott S, Trimbour R, Ramirez Flores RO, Argelaguet R, Saez-Rodriguez J (2023). Gene regulatory network inference in the era of single-cell multi-omics. Nat Rev Genet.

[18] Hu J, Zhang L, Xia H, Yan Y, Zhu X, Sun F, Sun L, Li S, Li D, Wang J, Han Y, Zhang J, Bian D, Yu H, Chen Y, Fan P, Ma Q, Jiang G, Wang C, Zhang P (2023). Tumor microenvironment remodeling after neoadjuvant immunotherapy in non-small cell lung cancer revealed by single-cell RNA sequencing. Genome Med.

[19] Li J, Wu C, Hu H, Qin G, Wu X, Bai F, Zhang J, Cai Y, Huang Y, Wang C, Yang J, Luan Y, Jiang Z, Ling J, Wu Z, Chen Y, Xie Z, Deng Y (2023). Remodeling of the immune and stromal cell compartment by PD-1 blockade in mismatch repair-deficient colorectal cancer. Cancer Cell.

[20] Bassez A, Vos H, Van Dyck L, Floris G, Arijs I, Desmedt C, Boeckx B, Vanden Bempt M, Nevelsteen I, Lambein K, Punie K, Neven P, Garg AD, Wildiers H, Qian J, Smeets A, Lambrechts D (2021). A single-cell map of intratumoral changes during anti-PD1 treatment of patients with breast cancer. Nat Med.

[21] Cho JW, Hong MH, Ha SJ, Kim YJ, Cho BC, Lee I, Kim HR (2020). Genome-wide identification of differentially methylated promoters and enhancers associated with response to anti-PD-1 therapy in non-small cell lung cancer. Exp Mol Med.

[22] Jung H, Kim HS, Kim JY, Sun JM, Ahn JS, Ahn MJ, Park K, Esteller M, Lee SH, Choi JK (2019). DNA methylation loss promotes immune evasion of tumours with high mutation and copy number load. Nat Commun.

[23] Kim JY, Choi JK, Jung H (2020). Genome-wide methylation patterns predict clinical benefit of immunotherapy in lung cancer. Clin Epigenetics.

[24] Sun D, Liu J, Zhou H, Shi M, Sun J, Zhao S, Chen G, Zhang Y, Zhou T, Ma Y, Zhao Y, Fang W, Zhao H, Huang Y, Yang Y, Zhang L (2023). Classification of Tumor Immune Microenvironment according to programmed death-ligand 1 expression and Immune Infiltration predicts response to Immunotherapy Plus Chemotherapy in Advanced patients with NSCLC. J Thorac Oncol off Publ Int Assoc Study Lung Cancer.

[25] Liu D, Schilling B, Liu D, Sucker A, Livingstone E, Jerby-Arnon L, Zimmer L, Gutzmer R, Satzger I, Loquai C, Grabbe S, Vokes N, Margolis CA, Conway J, He MX, Elmarakeby H, Dietlein F, Miao D, Tracy A, Gogas H, Goldinger SM, Utikal J, Blank CU, Rauschenberg R, von Bubnoff D, Krackhardt A, Weide B, Haferkamp S, Kiecker F, Izar B, Garraway L, Regev A, Flaherty K, Paschen A, Van Allen EM, Schadendorf D (2019). Integrative molecular and clinical modeling of clinical outcomes to PD1 blockade in patients with metastatic melanoma. Nat Med.

[26] Stuart T, Butler A, Hoffman P, Hafemeister C, Papalexi E, Mauck WM, Hao Y, Stoeckius M, Smibert P, Satija R (2019). Comprehensive Integration of Single-Cell Data. Cell.

[27] Wolock SL, Lopez R, Klein AM, Scrublet (2019). Computational identification of cell doublets in single-cell Transcriptomic Data. Cell Syst.

[28] Korsunsky I, Millard N, Fan J, Slowikowski K, Zhang F, Wei K, Baglaenko Y, Brenner M, Loh PR, Raychaudhuri S (2019). Fast, sensitive and accurate integration of single-cell data with Harmony. Nat Methods.

[29] Xing X, Yang F, Huang Q, Guo H, Li J, Qiu M, Bai F, Wang J (2021). Decoding the multicellular ecosystem of lung adenocarcinoma manifested as pulmonary subsolid nodules by single-cell RNA sequencing. Sci Adv.

[30] Aibar S, González-Blas CB, Moerman T, Huynh-Thu VA, Imrichova H, Hulselmans G, Rambow F, Marine JC, Geurts P, Aerts J, van den Oord J, Atak ZK, Wouters J, Aerts S (2017). SCENIC: single-cell regulatory network inference and clustering. Nat Methods.

[31] Cheng S, Li Z, Gao R, Xing B, Gao Y, Yang Y, Qin S, Zhang L, Ouyang H, Du P, Jiang L, Zhang B, Yang Y, Wang X, Ren X, Bei JX, Hu X, Bu Z, Ji J, Zhang Z (2021). A pan-cancer single-cell transcriptional atlas of tumor infiltrating myeloid cells. Cell.

[32] Fuxman Bass JI, Diallo A, Nelson J, Soto JM, Myers CL, Walhout AJM (2013). Using networks to measure similarity between genes: association index selection. Nat Methods.

[33] Andreatta M, Berenstein AJ, Carmona SJ (2022). scGate: marker-based purification of cell types from heterogeneous single-cell RNA-seq datasets. Bioinforma Oxf Engl.

[34] Kang JB, Nathan A, Weinand K, Zhang F, Millard N, Rumker L, Moody DB, Korsunsky I, Raychaudhuri S (2021). Efficient and precise single-cell reference atlas mapping with Symphony. Nat Commun.

[35] Zhang Q, He Y, Luo N, Patel SJ, Han Y, Gao R, Modak M, Carotta S, Haslinger C, Kind D, Peet GW, Zhong G, Lu S, Zhu W, Mao Y, Xiao M, Bergmann M, Hu X, Kerkar SP, Vogt AB, Pflanz S, Liu K, Peng J, Ren X, Zhang Z (2019). Landscape and Dynamics of Single Immune Cells in Hepatocellular Carcinoma. Cell.

[36] Zilionis R, Engblom C, Pfirschke C, Savova V, Zemmour D, Saatcioglu HD, Krishnan I, Maroni G, Meyerovitz CV, Kerwin CM, Choi S, Richards WG, Rienzo AD, Tenen DG, Bueno R, Levantini E, Pittet MJ, Klein AM (2019). Single cell transcriptomics of human and mouse lung cancers reveals conserved myeloid populations across individuals and species. Immunity.

[37] Desvergne B, Wahli W (1999). Peroxisome proliferator-activated receptors: nuclear control of metabolism. Endocr Rev.

[38] Liu C, Tate T, Batourina E, Truschel ST, Potter S, Adam M, Xiang T, Picard M, Reiley M, Schneider K, Tamargo M, Lu C, Chen X, He J, Kim H, Mendelsohn CL (2019). Pparg promotes differentiation and regulates mitochondrial gene expression in bladder epithelial cells. Nat Commun.

[39] Tate T, Xiang T, Wobker SE, Zhou M, Chen X, Kim H, Batourina E, Lin CS, Kim WY, Lu C, Mckiernan JM, Mendelsohn CL (2021). Pparg signaling controls bladder cancer subtype and immune exclusion. Nat Commun.

[40] Lu J, Liang T, Li P, Yin Q (2023). Regulatory effects of IRF4 on immune cells in the tumor microenvironment. Front Immunol.

[41] Alissafi T, Hatzioannou A, Legaki AI, Varveri A, Verginis P (2019). Balancing cancer immunotherapy and immune-related adverse events: the emerging role of regulatory T cells. J Autoimmun.

