# Supplementary figures and images for: Integrated analysis of single-cell and bulk RNA sequencing data reveals a myeloid cell-related regulon predicting neoadjuvant immunotherapy response across cancers
Source: J Transl Med. 2024 May 21;22:486. doi: 10.1186/s12967-024-05123-9 (PMC11110189; doi:10.1186/s12967-024-05123-9)

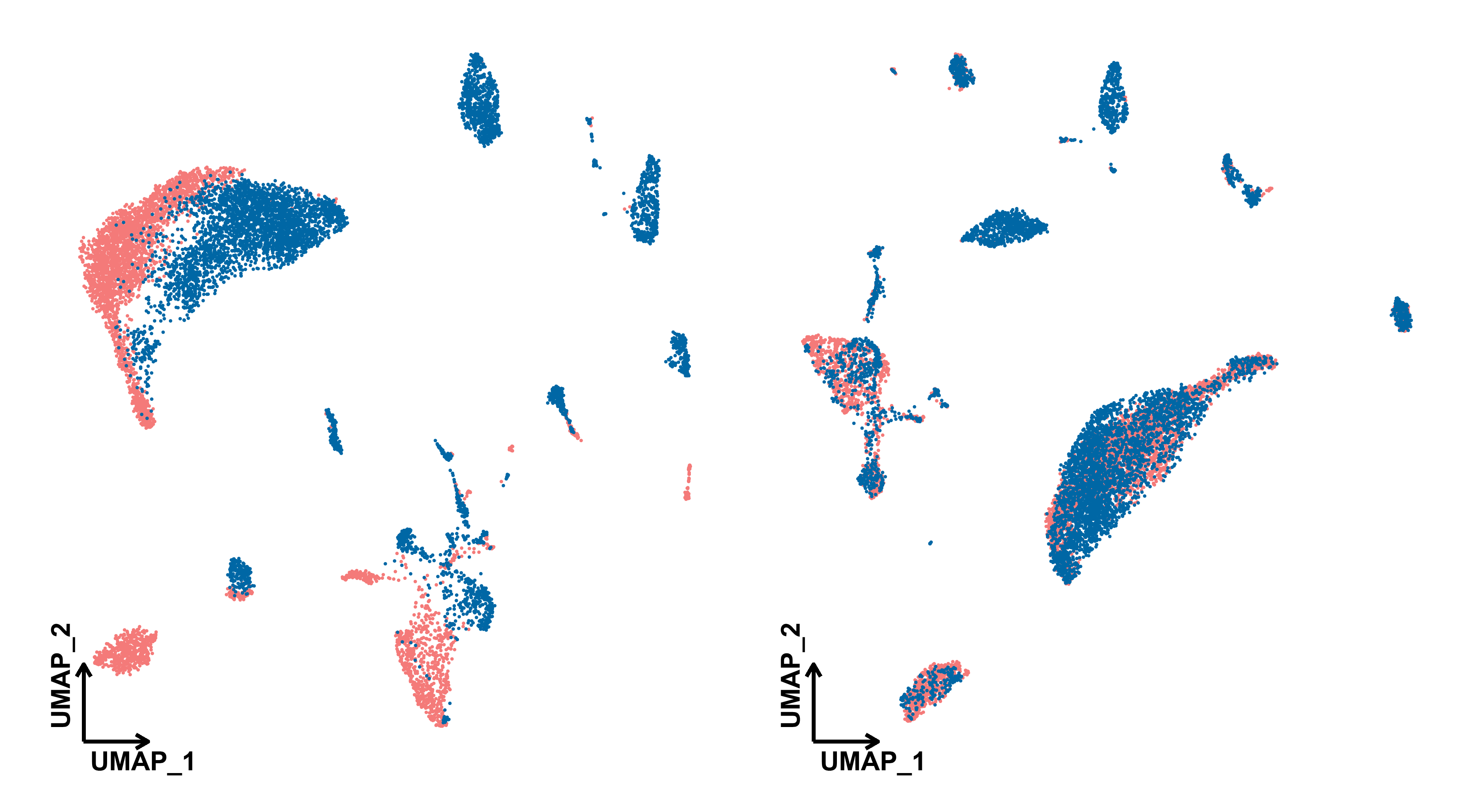

Supplement: Supplementary file 2 — Supplementary Material 2 [file 12967_2024_5123_MOESM2_ESM.png]

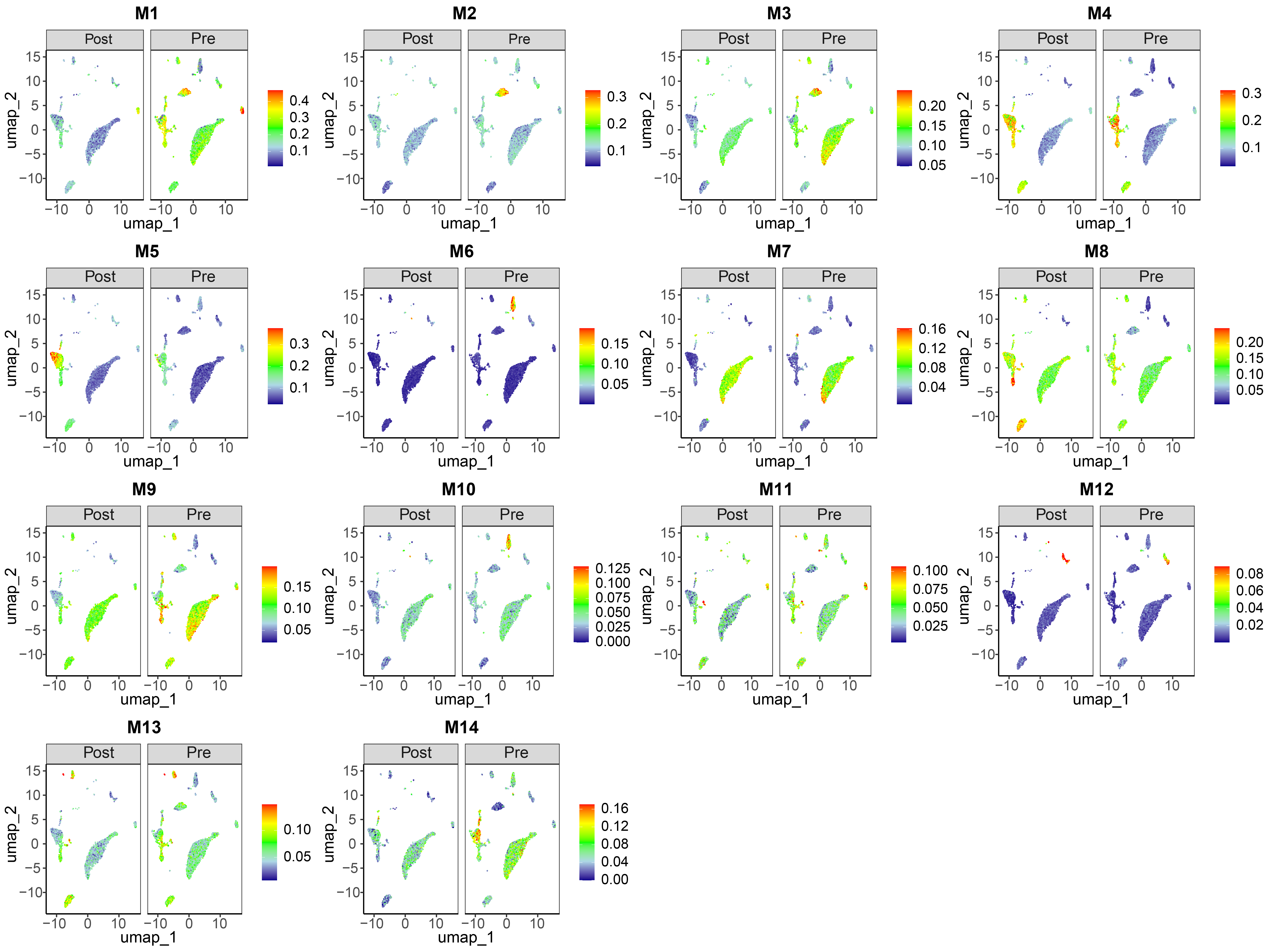

Supplement: Supplementary file 3 — Supplementary Material 3 [file 12967_2024_5123_MOESM3_ESM.png]

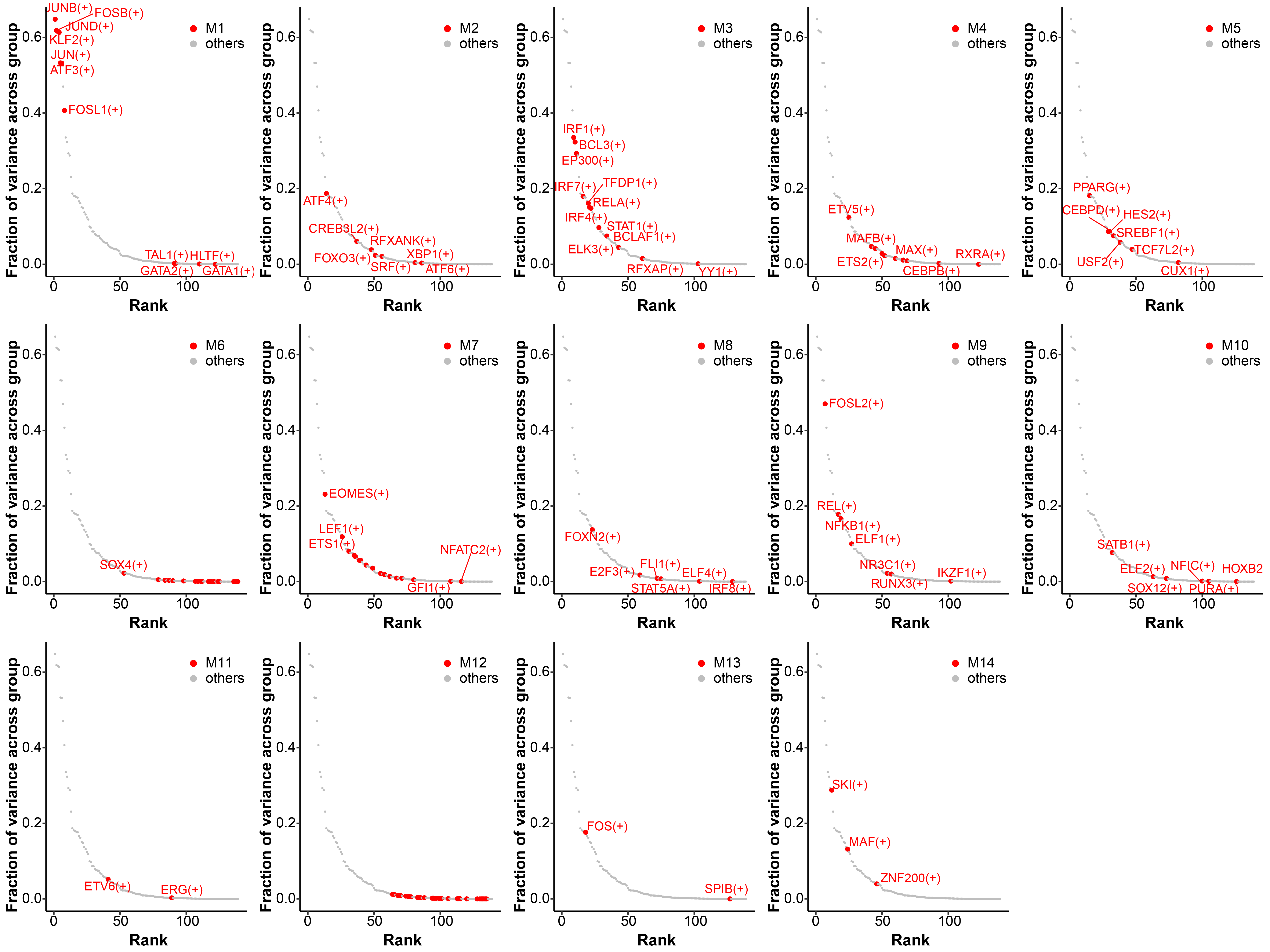

Supplement: Supplementary file 4 — Supplementary Material 4 [file 12967_2024_5123_MOESM4_ESM.png]

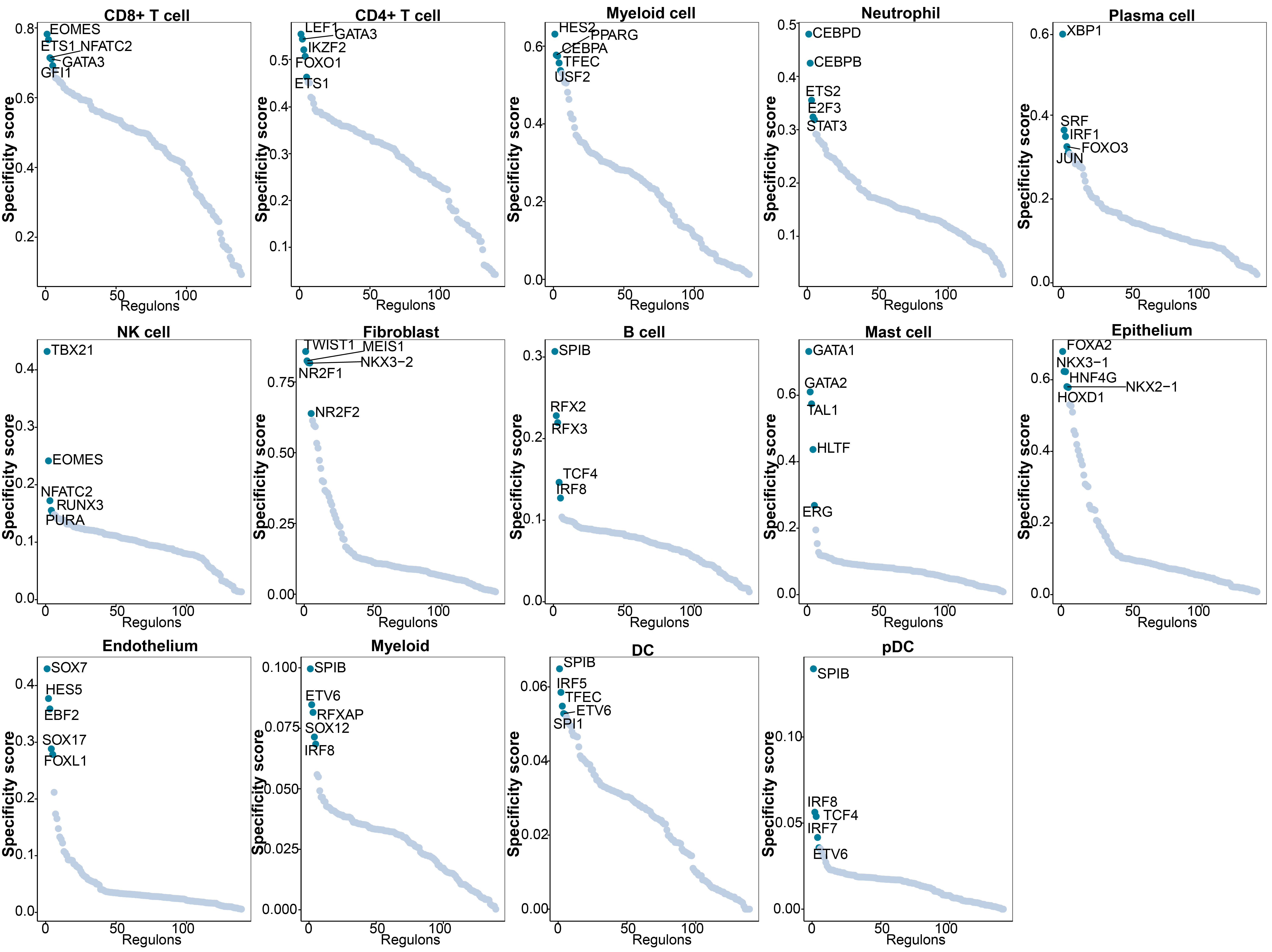

Supplement: Supplementary file 5 — Supplementary Material 5 [file 12967_2024_5123_MOESM5_ESM.png]

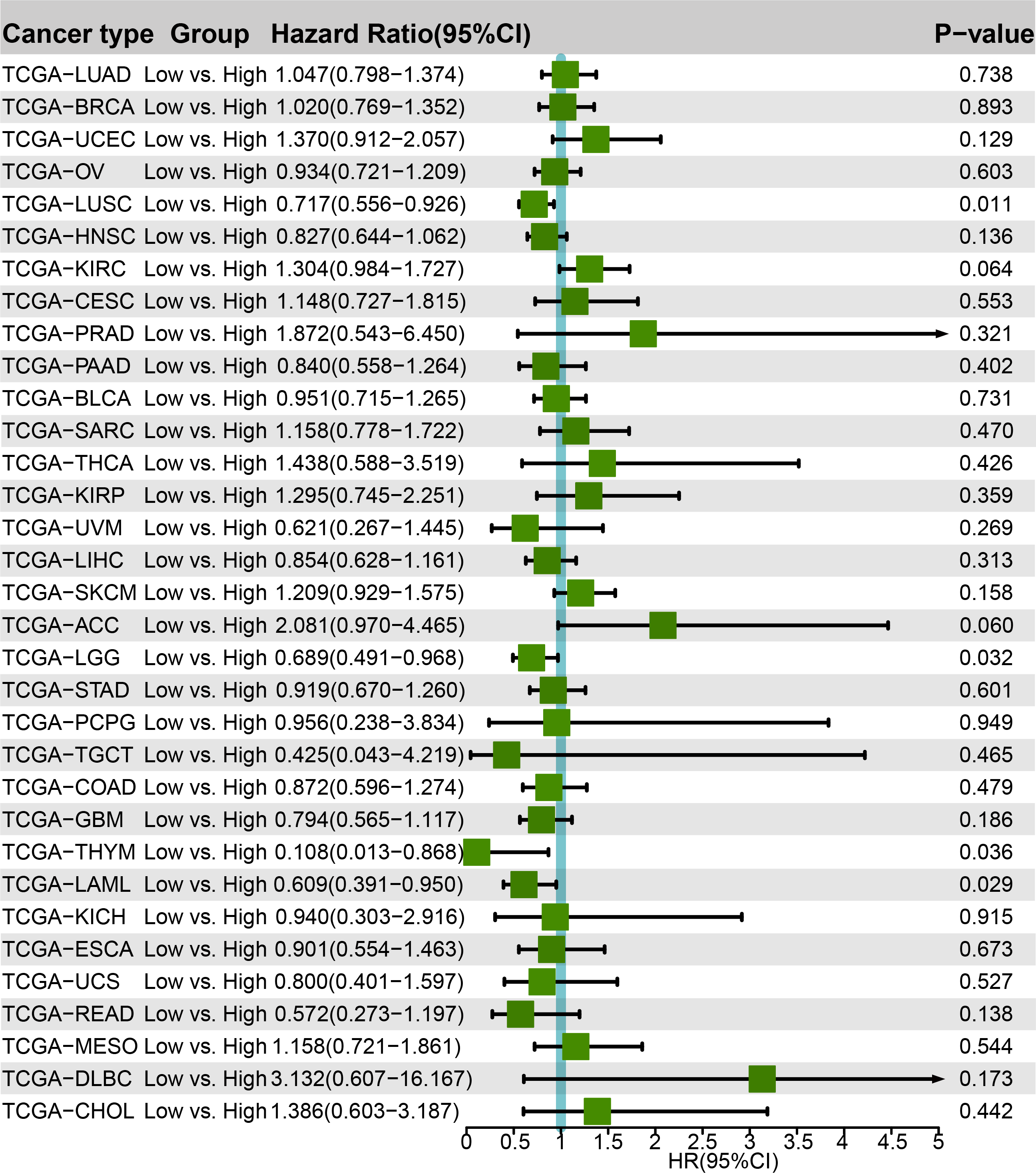

Supplement: Supplementary file 6 — Supplementary Material 6 [file 12967_2024_5123_MOESM6_ESM.png]

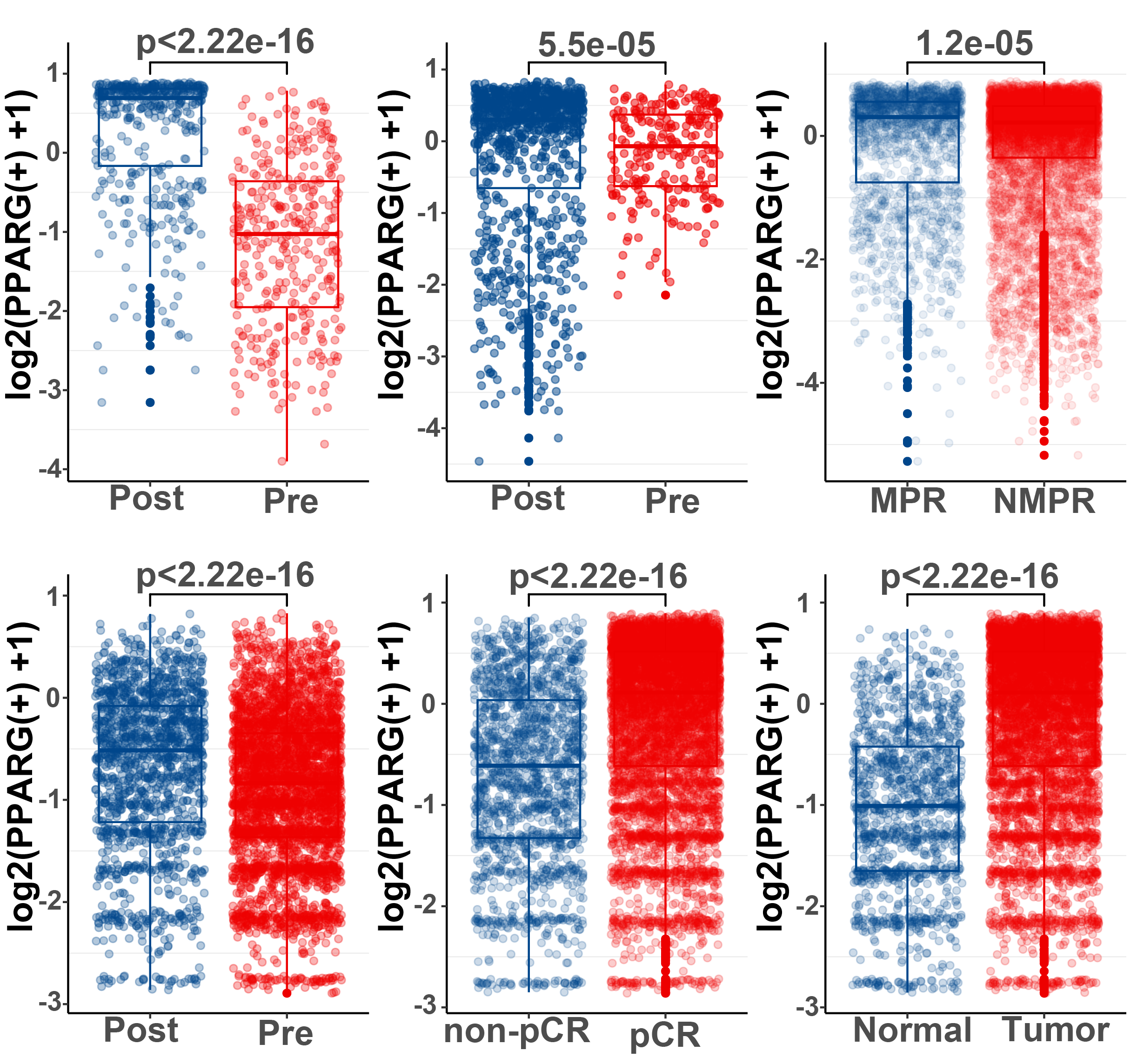

Supplement: Supplementary file 7 — Supplementary Material 7 [file 12967_2024_5123_MOESM7_ESM.png]
