# Supplementary material for: Integrated analysis of single-cell and bulk RNA sequencing data reveals a myeloid cell-related regulon predicting neoadjuvant immunotherapy response across cancers
Source: J Transl Med. 2024 May 21;22:486. doi: 10.1186/s12967-024-05123-9 (PMC11110189; doi:10.1186/s12967-024-05123-9)
Supplement: Supplementary file 8 — Supplementary Material 8 [file 12967_2024_5123_MOESM8_ESM.docx]

**Supplementary Fig. 1** UMAP for all single cells of GSE207422 patient 08 and patient 06 before (left) and after (right) removal of the batch effect by using Harmony.

**Supplementary Fig. 2** UMAP for all single cells from GSE207422 patient 08 and patient 06 in modules M1-M14 based on the average regulon activity score (RAS).

**Supplementary Fig. 3** Regulons ranked by analysing the variance components in modules M1-M14 (red dots).

**Supplementary Fig. 4** Rank for regulons in the 14 major cell types based on regulon specificity score (RSS).

**Supplementary Fig. 5** TCGA pan-cancer univariable Cox regression analysis results based on the PPARG regulon.

**Supplementary Fig. 6** Box plots of PPARG regulon scores by GSVA in different groups. A. GSE207422 pre- and posttreatment groups (patient 08 and patient 06); B. GSE207422 pretreatment and posttreatment achieving MPR groups (patient 08 and patient 06).
